# Variant Alleles of the *ESR1, PPARG, HMGA2*, and *MTHFR* Genes Are Associated With Polycystic Ovary Syndrome Risk in a Chinese Population: A Case-Control Study

**DOI:** 10.3389/fendo.2018.00504

**Published:** 2018-08-30

**Authors:** Xianting Jiao, Weiwei Chen, Jun Zhang, Weiye Wang, Junjiao Song, Dan Chen, Wenting Zhu, Yuhua Shi, Xiaodan Yu

**Affiliations:** ^1^Department of Developmental and Behavioral Pediatrics, Shanghai Children's Medical Center, Pediatric Translation Medicine Institute, Shanghai JiaoTong University School of Medicine, Shanghai, China; ^2^MOE-Shanghai Key Laboratory of Children's Environmental Health, Xinhua Hospital Affiliated to Shanghai Jiao Tong University School of Medicine, Shanghai, China; ^3^Center for Reproductive Medicine, Shandong University, Jinan, China

**Keywords:** polycystic ovary syndrome, single-nucleotide polymorphism, infertility, endocrinopathy, variant alleles

## Abstract

Polycystic ovary syndrome (PCOS) is the most common endocrinopathy in women of reproductive age, with a prevalence of 6–8%. Although the etiology of PCOS has been investigated extensively, the association between genetic predisposition and PCOS risk is largely unknown. In this study, we genotyped 63 SNPs in 10 genes among 361 PCOS patients and 331 healthy controls in a Chinese Han population. The following variant alleles were significantly associated with decreased PCOS risk: *ESR1* rs9340799 (*P* = 0.000), *PPARG* rs709154 (*P* = 0.013), and rs1151996 (*P* = 0.013), *HMGA2* rs2272046 (*P* = 0.000), *MTHFR* rs1801133 (*P* = 0.000). Accordingly, the following genotypes at various loci were associated with reduced PCOS risk: GA genotype at rs9340799 (*P* < 0.0001) in *ESR1*, TA genotype at rs709154(*P* < 0.0001) in *PPARG* and CA genotype at rs2272046 (*P* < 0.0001) in *HMGA2*. Moreover, GA genotype at rs1999805 (*P* = 0.013) in *ESR1* and TT genotype at rs1801133 in *MTHFR* (*P* < 0.0001) correlated with elevated PCOS risk. Furthermore, haplotype analysis revealed significant differences in haplotype distributions of *CYP11A1, ESR2* and *PPARG* gene between cases and controls. In addition to confirming that *ESR1* rs9340799, *HMGA2* rs2272046 and *MTHFR* rs1801133 are related to the risk of PCOS, these findings also provide the first evidence that *PPARG* rs709154 and *ESR1* rs1999805 are significantly associated with PCOS risk in a Chinese population. Further functional studies are warranted to elucidate the underlying biological mechanisms.

## Introduction

Polycystic ovary syndrome (PCOS) is the most common endocrinopathy in women of reproductive age, with a prevalence of 6–8% (1). This syndrome is characterized by hyperandrogenism, amenorrhea or oligomenorrhea and polycystic ovaries (2, 3). Women with PCOS are potentially at elevated risk of multiple diseases and disorders, including infertility (4), insulin resistance (5), obesity (6), premature carotid arteriosclerosis (7), type 2 diabetes mellitus (8), and mood disorders, such as depression and anxiety (9, 10). Epidemiological studies suggest that androgen excess, lifestyle (11), ovulatory dysfunction, alteration in intrauterine environment (12), adipose tissue dysfunction and gonadotropin abnormalities can contribute to PCOS risk (13). The etiology of PCOS is complicated and not well elucidated.

A substantial body of evidence has implicated various genetic factors in PCOS development (14). Related studies have been conducted on at least 70 genes, which roles are involved in the main process of this disease, such as steroid synthesis (*CYP11A1* and *CYP19A1*) (15), steroid action (*ESR1, ESR2*, and *PGR*) (16), lipid metabolism (*PPARG* and *MTHFR*) (17–19), insulin action (*HMGA2*) (20) and embryonic development (*SUMO1P1*) (21, 22) have been implicated in PCOS. However, the heterogeneity and generalizability of these genetic associations are not enough to explain clearly the considerable genetic susceptibility for this endocrine-metabolic disorder. For example, *FSHR* rs6166 was associated with increased PCOS risk in a study of 377 Chinese PCOS patients and 388 age-matched healthy controls (23), but not in a study in a different ethnic population (24). *ESR1* rs9340799 has been associated with increased PCOS risk in a Pakistani population (25), but not in a Brazilian population (26).

Therefore, finding as many single-nucleotide polymorphisms (SNPs) as possible through population studies will provide valuable assistance to clinicians and patients. Except for the previous reported genes which are involved in the pathogenesis of PCOS, morphological changes in the ovary is a key part in the development of PCOS. Related studies have proved that *LAMC1* can promote the development of follicles (27) and some SNP polymorphisms were significantly associated with premature ovarian failure (POF) (28). Because of the role of follicular development in the development of PCOS, the association between *LAMC1* polymorphism and PCOS risk deserved studying.

Considering the important role of genetic factors in this pathogenesis of PCOS, the aim of the study was to discover some genetic variations associated with these abnormal pathological mechanisms. So we have chosen several sites in *CYP11A1, CYP19A1, ESR1, ESR2, PGR, PPARG, LAMC1, HMGA2, MTHFR*, and *SUMO1P1* as our research object according to the functional description in the literature. We genotyped for 63 SNPs including 40 which had never been assessed for their potential association with PCOS in 10 known pathogenicity-causing genes by a case-control study of 361 PCOS cases and 331 controls in a Chinese population.

## Materials and methods

### Sample collection

A total of 361 PCOS patients and 331 controls, all of Han Chinese ethnicity, were included in this multi-center study. Subjects were recruited between January 2015 and February 2017 at the Reproductive Hospital Affiliated to Shandong University, at the Women's Hospital of the School of Medicine of Zhejiang University and at the Renji Hospital Affiliated to Shanghai Jiao Tong University. PCOS was diagnosed based on the NIH criteria [National Institutes of Health/National Institute of Child Health and Human Development (NIH/NICHD) in April 1990], including biochemical and/or clinical hyperandrogenism and ovulatory dysfunction, after the exclusion of related or other disorders (1). This study was approved by the Shanghai Xinhua Hospital Research Ethics Board and all subjects gave their informed consent for inclusion before they participated in the study. Women were excluded if they had been diagnosed with 21-hydroxylase-deficiency, androgen-producing tumors, hyperprolactinemia, non-classical adrenal hyperplasia, Cushing's syndrome, and active thyroid disease, since these conditions likely affect reproductive physiology (19, 29). All the controls were defined as women with normal hormonal status and regular menstrual cycles at intervals of 28–35 days. Information on participants was collected from medical records.

### Biochemical and hormonal analyses

Peripheral blood samples were taken from participants in a fasting state on days 3–5 of the menstrual cycle. Blood samples were collected into EDTA anticoagulant tubes, centrifuged, and stored at −80°C. Levels of luteinizing hormone (LH), follicle-stimulating hormone (FSH), testosterone (T), and estradiol (E_2_) were measured using a chemiluminescent analyzer (Beckman Coulter, Fullerton, CA, USA). The limit of detection (LOD) of LH, FSH, T and E2 was 0.1 IU/L, 0.1 IU/L, 0.1 nmol/L and 0.1 pg/ml respectively. They intra- and inter-assay coefficients of variations (CV) were < 6% and < 10%, respectively.

### SNP selection and genotyping

A total of 63 SNPs were selected in the following 10 genes, mainly based on literature review: *CYP11A1, CYP19A1, ESR1, ESR2, PGR, PPARG, MTHFR, HMGA2, LAMC1*, and *SUMP1O1* (21). Five of the SNPs were 3′-flanking variants; 57 SNPs, intronic variants; 3 SNPs in exons, non-synonymous variants; 3 SNPs in exons, synonymous variants; and 2 SNPs near 5′-flanking regions. Genomic DNA was extracted from the buffy coat using DNA isolation kits (TIANGEN, China) according to the manufacturer's protocol. Genotyping was carried out using polymerase chain reaction ligation detection reaction (PCR-LDR). Genotyping was performed by technicians blinded to case or control status. In addition, 5% of samples were re-genotyped, and the results agreed 100% with the first results. PCRs were carried out on an ABI 7300 (Applied Biosystem, Foster City, CA, USA) in a total volume of 20 μl including 20 ng genomic DNA, 2.5 mM MgCl2, 0.2 mM dNTPs, 0.5 μM each primer, 1 × PCR Buffer and 1 U Hot-Start Taq DNA polymerase (QIAgen). Cycling parameters were set as follows: 95°C for 10 min, 38 cycles at 94°C for 15 s, 60°C for 1.5 min, 72°C for 60 s, followed by 72°C for 10 min. The ligation reaction was carried out in a final volume of 20 μl containing 2 μl template DNA, 1 μl 10 × ligation buffer, 20 U Taq DNA ligase and 1 pmol of each discriminating probe (New England Biolabs, USA). The LDR parameters were as follows: 94°C for 1.5 min, 35 cycles at 94°C for 15 s and 58°C for 1.5 min. Following the LDR reaction, 1 μl LDR reaction product was mixed with 1 μl loading buffer as well as 1 μl ROX. The mixture was then analyzed using the ABI 7900HT (Applied Biosystems).

### Statistical analysis

Hormone levels, differences in age and body mass index (BMI) between controls and cases were evaluated using Student's *t*-test. The fit of results for each SNP in controls was assessed against expectations based on Hardy-Weinberg equilibrium (HWE) using the χ^2^-test. Associations between selected polymorphisms and PCOS were assessed using unconditional logistic regression with adjustment for BMI and age.

Pairwise linkage disequilibrium (LD) among the selected SNPs was examined using Lewontin's standardized coefficient D' and LD coefficient *r*^2^ (30). Haplotype blocks were defined by the method of (31) in the publicly available Haploview software (www.broadinstitute.org/haploview/downloads) using default settings: the confidence interval for a strong LD was minimal for upper 0.98 and low 0.7 and maximal for a strong recombination of 0.9, and a fraction of strong LD in informative comparisons was at least 0.95. Haplotype analysis was performed using the SNP stats tool (www.snpstats.net/snpstats/start.htm?q=snpstats/start.htm), with adjustment for age and BMI where appropriate. Multiple test corrections were performed for each gene using Bonferroni correction. All statistical analyses were performed using SAS 9.4 software and R. *P* < 0.05 was considered statistically significant.

## Results

### Characteristics of the participants

The characteristics of the participants are shown in Table 1. The age of cases ranged from 19 to 40 years (mean ± SD, 28.1 ± 3.7); the age of controls, from 22 to 45 years (28.4 ± 4.2, *P* = 0.041). As expected, levels of LH, testosterone and BMI in the PCOS group were significantly higher than that in healthy controls.

**Table 1:** Characteristics of participants.

**Characteristic**	**Controls (*n* = 331)**	**Cases (*n* = 361)**	***P^a^***
Age (years)	28.4 ± 4.2	28.1 ± 3.7	0.364
BMI (kg/m^2^)	21.5 ± 3.4	24.9 ± 4.3	<**0.0001**
< 24	282 (85.2%)	169 (46.8)	
≥24	49 (14.8%)	192 (53.2)	
LH (IU/L)	4.1 ± 4.7	7.0 ± 5.8	<**0.0001**
FSH (IU/L)	4.1 ± 3.6	5.4 ± 2.6	**0.0002**
LH/FSH	0.6 ± 0.7	1.2 ± 1.1	<**0.0001**
T (nmol/L)	2.8 ± 3.1	23.7 ± 23.4	<**0.0001**
E_2_ (pg/ml)	88.2 ± 75.3	104.7 ± 165.8	0.775

*Continuous data were expressed as mean ± SD; BMI, body mass index; LH, luteinizing hormone; FSH, follicle-stimulating hormone; T, testosterone; E_2_, estradiol*.

a *Boldface indicates P < 0.05*.

### Association between individual SNPs and PCOS risk

Results on minor allele frequencies and HWE at selected SNPs are shown in Table 2. Among the 63 polymorphisms, five deviated from HWE (*P* < 0.05): rs1801132, rs1884051, rs722208, rs932477, and rs728524. Minor allele frequencies of 0 were observed for the following five SNPs: rs1913474, rs1042839, rs480851, rs1805192, and rs709150. These 10 SNPs were excluded from subsequent analysis. Among 53 polymorphic locus analysis, we found that the following variant alleles were significantly associated with decreased PCOS risk: The A>G allele distributions at rs9340799 in *ESR1* (*P* = 0.000), the A>T at rs709154 (*P* = 0.013), and A>C at rs1151996 (*P* = 0.013) in *PPARG*. the A>C at rs2272046 in *HMGA2* (*P* = 0.000), and the T>C at rs1801133 in *MTHFR* (*P* = 0.000, Table 3). Further we detected the following genotype distribution were associated with decreased PCOS risk: GA genotype at rs9340799 (*P* < 0.0001) in *ESR1*, TA genotype at rs709154 (*P* < 0.0001) in *PPARG*, CA genotype at rs2272046 (*P* < 0.0001) in *HMGA2*. Moreover, GA genotype at rs1999805 (*P* = 0.013) in *ESR1* and TT genotype at rs1801133 in *MTHFR* (*P* < 0.0001) correlated with elevated PCOS risk (Table 4). We conducted a stratified analysis of risk-related loci by BMI (BMI < 24, BMI ≥ 24; Supplementary Table 1). Finally we found that BMI may not affect the risk of rs9340799 and rs1999805 in *ESR1*, rs2272046 in *HMGA2*, rs1801133 in *MTHFR*, rs709154 in *PPARG* gene and PCOS.

**Table 2:** Information about minor allele frequency and Hardy-Weinberg equilibrium at selected SNPs.

**Gene**	**SNP**	**Genomic position**	**Position in gene**	**MAF**	**HWE**	**Genotyping**
						**Rate (%)**
*CYP11A1*	rs9806234	74663033	Intron	0.35	0.19	96.3
	rs11632698	74637867	Intron	0.25	0.47	99.2
	rs11638442	74634899	Intron	0.25	0.62	99.6
	rs16968478	74662811	Intron	0.45	0.12	97.2
	rs1843090	74643034	Intron	0.41	0.69	96.5
	rs2073475	74661894	5′ near gene	0.41	0.56	97.8
	rs2279357	74630623	Intron	0.41	0.56	97.4
	rs4887139	74661945	5′ near gene	0.1	1.00	97.2
	rs7173655	74632362	Intron	0.4	0.43	97.9
*CYP19A1*	rs2414096	51529779	Intron	0.47	0.70	96.5
	rs700518	51529112	Synonymous exon	0.47	1.00	96.7
*ESR1*	rs1709183	152193996	Intron	0.41	0.56	98.4
	rs1801132	152265522	Synonymous exon	0.48	**0.00**^b^	98.4
	rs1884051	152283279	Intron	0.47	**0.01**^b^	98.2
	rs2228480	152420095	Intron	0.26	0.06	97.9
	rs2234693	152163335	Intron	0.41	0.69	98.9
	rs3020314	152270672	Intron	0.14	0.07	100.0
	rs3778082	152387664	Intron	0.33	0.52	97.4
	rs3778099	152418575	Intron	0.37	0.22	97.2
	rs3798573	152389362	Intron	0.34	0.40	96.9
	rs3798577	152421130	Intron	0.46	0.33	98.4
	rs722208	152322885	Intron	0.49	**0.01**^b^	97.8
	rs851982	152024985	Intron	0.13	0.60	96.7
	rs9322331	152162317	Intron	0.03	1.00	98.8
	rs932477	152304596	Intron	0.39	**0.01**^b^	99.4
	rs9340799	152163381	Intron	0.19	0.61	96.9
	rs1913474	152208722	Intron	**0.00**^a^	1.00	23.4
	rs1999805	152068364	Intron	0.33	0.23	97.4
	rs728524	152303437	Intron	0.21	**0.02**^b^	97.9
*ESR2*	rs1255998	64693871	3′ utr	0.48	0.55	96.3
	rs928554	64694195	3′ utr	0.66	0.31	97.9
	rs960070	64745179	Intron	0.25	1.00	94.3
	rs1152579	64695087	Intron	0.3	0.83	96.7
	rs944459	64699358	Intron	0.27	0.10	97.9
*PGR*	rs1042839	100922202	Synonymous exon	**0.00**^a^	1.00	100
	rs471767	100905297	3′ utr	0.05	1.00	99.8
	rs484389	100909809	3′ utr	0.16	0.26	96.7
	rs508653	100962200	Intron	0.15	0.06	98.9
	rs547378	100922939	Intron	0.16	0.26	99.4
	rs561650	100915894	Intron	0.14	0.69	98.4
	rs566351	100985014	Intron	0.15	0.42	98.2
	rs572483	100967572	Intron	0.15	0.07	97.6
	rs613120	100974278	Intron	0.15	0.06	98.6
	rs480851	100976191	Intron	**0.00**^a^	1.00	98.9
	rs500760	100909991	Synonymous exon	0.16	0.26	97.3
	rs608995	100905733	3′ utr	0.16	0.26	96.7
*PPARG*	rs1801282	12393125	Intron	0.06	1.00	99.1
	rs1805192	12421238	Non-synonymous exon	**0.00**^a^	1.00	100
	rs709149	12450354	Intron	0.43	0.51	95.5
	rs709150	12451337	Intron	**0.00**^a^	1.00	28.2
	rs709151	12454999	Intron	0.39	0.18	98.2
	rs709154	12456834	Intron	0.36	0.25	98.6
	rs1151996	12445807	Intron	0.47	1.00	98.2
*LAMC1*	rs1413390	183096634	Intron	0.45	0.55	95.9
	rs1537520	183084229	Intron	0.45	0.43	98.2
	rs20558	183094547	Non-synonymous exon	0.47	0.24	98.6
	rs2296293	183095477	Intron	0.45	0.16	96.5
	rs2296300	183099701	Intron	0.45	0.33	98.8
	rs6424888	183085696	Intron	0.45	0.33	98.8
	rs6672093	183079853	Intron	0.47	0.43	98.6
*HMGA2*	rs2272046	66224461	Intron	0.07	0.21	99.8
*MTHFR*	rs1801133	11856378	Non-synonymous exon	0.47	0.42	92.7
*SUMO1P1*	rs6022786	52447303	Intron	0.33	0.54	96.7

a *Boldface is used for minor allele frequencies of 0*.

b *Boldface for deviation from HWE*.

*SNP, single-nucleotide polymorphism; MAF, minor allele frequency; HWE, Hardy-Weinberg equilibrium, HWE P value in the control group*.

**Table 3:** Allele frequencies of the selected genetic variants analyzed in PCOS patients and controls.

**No**.	**Gene**	**SNP**	**Base**	**OR (95%CI)**	***P*^a^**	***P_*ajusted*_***
			**Change**			
1	*CYP11A1*	rs9806234	G>A	1.15(0.88,1.50)	0.299	0.474
2		rs11632698	A>G	0.87(0.66,1.14)	0.311	0.479
3		rs11638442	C>G	0.89(0.67,1.17)	0.393	0.593
4		rs16968478	A>G	0.97(0.76,1.22)	0.779	0.874
5		rs1843090	G>A	0.91(0.71,1.17)	0.475	0.684
6		rs2073475	C>T	0.77(0.60,0.99)	0.042	0.150
7		rs2279357	C>T	0.91(0.71,1.17)	0.480	0.685
8		rs4887139	A>G	2.10(1.36,3.26)	0.001	0.068
9		rs7173655	C>T	0.97(0.75,1.26)	0.832	0.908
10	*CYP19A1*	rs2414096	G>A	1.15(0.91,1.45)	0.251	0.419
11		rs700518	T>C	1.06(0.83,1.34)	0.664	0.783
12	*ESR1*	rs1709183	C>T	1.02(0.79,1.31)	0.900	0.955
13		rs2228480	G>A	1.46(1.08,1.98)	0.013	0.079
14		rs2234693	T>C	1.03(0.80,1.32)	0.840	0.910
15		rs3020314	C>T	0.71(0.49,1.04)	0.081	0.218
16		rs3778082	G>A	0.76(0.58,1.00)	0.053	0.166
17		rs3778099	T>C	0.92(0.68,1.25)	0.594	0.740
18		rs3798573	A>G	0.74(0.57,0.97)	0.027	0.121
19		rs3798577	T>C	0.67(0.51,0.87)	0.003	0.124
20		rs851982	T>C	1.68(1.14,2.47)	0.008	0.218
21		rs9322331	C>T	1.46(0.70,3.04)	0.314	0.479
22		rs9340799	A>G	0.50(0.37,0.67)	0.000	**0.000**
23		rs1999805	G>A	1.22(0.95,1.57)	0.123	0.280
24	*ESR2*	rs1255998	C>G	1.05(0.83,1.33)	0.656	0.783
25		rs928554	T>C	0.83(0.63,1.08)	0.159	0.324
26		rs960070	G>C	1.36(1.01,1.84)	0.042	0.150
27		rs1152579	G>A	0.75(0.58,0.98)	0.036	0.135
28		rs944459	C>T	0.90(0.69,1.17)	0.430	0.643
29	*PGR*	rs471767	A>G	1.19(0.68,2.09)	0.538	0.716
30		rs484389	A>G	1.46(1.04,2.06)	0.030	0.124
31		rs508653	C>A	1.35(0.96,1.91)	0.085	0.222
32		rs547378	G>A	1.49(1.06,2.09)	0.022	0.102
33		rs561650	T>C	1.50(1.04,2.17)	0.031	0.125
34		rs566351	C>T	1.42(0.99,2.01)	0.053	0.166
35		rs572483	T>C	1.31(0.93,1.84)	0.117	0.274
36		rs613120	A>G	1.29(0.92,1.81)	0.143	0.299
37		rs500760	T>C	1.45(1.03,2.04)	0.032	0.126
38		rs608995	A>T	1.50(1.07,2.11)	0.019	0.095
39	*PPARG*	rs1801282	C>G	0.56(0.35,0.91)	0.020	0.095
40		rs709149	G>A	0.87(0.66,1.14)	0.311	0.479
41		rs709151	C>T	0.72(0.56,0.94)	0.017	0.092
42		rs709154	A>T	0.65(0.50,0.85)	0.001	**0.013**
43		rs1151996	A>C	0.66(0.51,0.85)	0.001	**0.013**
44	*LAMC1*	rs1413390	A>G	1.19(0.92,1.53)	0.191	0.346
45		rs1537520	T>C	1.19(0.92,1.55)	0.180	0.340
46		rs20558	C>T	1.18(0.91,1.53)	0.209	0.361
47		rs2296293	A>G	1.26(0.97,1.65)	0.082	0.218
48		rs2296300	A>G	1.18(0.91,1.53)	0.213	0.363
49		rs6424888	G>A	1.18(0.91,1.53)	0.204	0.356
50		rs6672093	C>T	1.15(0.89,1.49)	0.274	0.443
51	*HMGA2*	rs2272046	A>C	0.28(0.18,0.43)	0.000	**0.000**
52	*MTHFR*	rs1801133	T>C	0.62(0.49,0.80)	0.000	**0.000**
53	*SUMO1P1*	rs6022786	G>A	0.74(0.57,0.95)	0.019	0.095

*Boldface indicates P < 0.05; CI, confidence interval; OR, odds ratio; SNP, single-nucleotide polymorphism; PCOS, polycystic ovarian syndrome*.

a *P-value from unconditional logistic regression with adjustment for BMI and age*.

*P_ajusted_ were performed for each gene using Bonferroni correction*.

**Table 4:** Genotype frequencies of selected polymorphisms analyzed in PCOS patients and controls.

**Gene**	**SNP**	**Controls**	**Cases**	**OR (95%CI)**	***P*^a^**	***P_*ajusted*_***
		**(*n* = 331)**	**(*n* = 361)**			
*CYP11A1*	rs9806234					
	G/G	124 (40.0)	139 (40.1)	Reference		
	G/A	156(50.3)	167 (48.1)	1.02(0.71–1.46)	0.926	0.974
	A/A	30 (9.7)	41 (11.8)	1.50(0.83–2.69)	0.178	0.340
	rs11632698					
	A/A	160 (50.2)	192 (53.6)	Reference		
	A/G	135 (42.3)	152 (42.5)	0.96 (0.68–1.36)	0.830	0.908
	G/G	24 (7.5)	14 (3.9)	0.60 (0.28–1.25)	0.170	0.340
	rs11638442					
	C/C	160 (50.8)	194 (53.7)	Reference		
	C/G	131 (41.6)	153 (42.4)	1.01 (0.71–1.44)	0.943	0.974
	G/G	24 (7.6)	14 (3.9)	0.59 (0.28–1.23)	0.158	0.324
	rs16968478					
	A/A	67 (20.6)	104 (29.7)	Reference		
	G/A	202 (62.0)	190 (54.3)	0.59 (0.39–0.88)	0.010	0.068
	G/G	57 (17.5)	56 (16.0)	0.55 (0.32–0.94)	0.028	0.122
	rs1843090					
	G/G	102 (32.5)	125 (35.7)	Reference		
	G/A	162 (51.6)	172 (49.1)	0.81 (0.56–1.18)	0.266	0.435
	A/A	50 (15.9)	53 (15.1)	0.89 (0.53–1.48)	0.652	0.783
	rs2073475					
	C/C	92 (28.3)	122 (34.7)	Reference		
	C/T	181 (55.7)	182 (51.7)	0.76 (0.52–1.10)	0.142	0.299
	T/T	52 (16.0)	48 (13.6)	0.60 (0.35–1.01)	0.055	0.169
	rs2279357					
	C/C	102 (30.8)	119 (34.1)	Reference		
	T/C	179 (54.1)	179 (51.3)	0.80 (0.55–1.16)	0.243	0.410
	T/T	50 (15.1)	51 (14.6)	0.89 (0.53–1.50)	0.675	0.785
	rs4887139					
	A/A	280 (87.2)	278 (79.0)	Reference		
	G/A	41 (12.8)	69 (19.6)	1.91 (1.21–3.04)	0.006	0.055
	G/G	0 (0.0)	5 (1.4)	NA	0.977	0.980
	rs7173655					
	C/C	101 (30.6)	113 (32.1)	Reference		
	C/T	186 (56.4)	189 (53.7)	0.95 (0.65–1.38)	0.777	0.874
	T/T	43 (13.0)	50 (14.2)	0.96 (0.56–1.65)	0.888	0.948
*CYP19A1*	rs2414096					
	G/G	106 (34.0)	99 (28.3)	Reference		
	G/A	131 (42.0)	174 (49.7)	1.40 (0.94–2.08)	0.100	0.249
	A/A	75 (24.0)	77 (22.0)	1.28 (0.80–2.04)	0.304	0.477
	rs700518					
	T/T	90 (29.9)	100 (28.4)	Reference		
	T/C	143 (47.5)	177 (50.3)	1.05 (0.70–1.58)	0.801	0.892
	C/C	68 (22.6)	75 (21.3)	1.11 (0.68–1.81)	0.668	0.783
*ESR1*	rs1709183					
	C/C	97 (29.5)	121 (34.1)	Reference		
	T/C	189 (57.4)	178 (50.1)	0.75 (0.52–1.09)	0.133	0.294
	T/T	43 (13.1)	56 (15.8)	1.22 (0.72–2.08)	0.460	0.675
	rs2228480					
	G/G	194 (60.8)	186 (52.4)	Reference		
	G/A	116 (36.4)	153 (43.1)	1.46 (1.03–2.07)	0.034	0.130
	A/A	9 (2.8)	16 (4.5)	2.18 (0.86–5.55)	0.102	0.250
	rs2234693					
	T/T	102 (32.5)	111 (30.9)	Reference		
	T/C	158 (50.3)	198 (55.2)	1.11 (0.76–1.63)	0.576	0.735
	C/C	54 (17.2)	50 (13.9)	1.02 (0.61–1.71)	0.941	0.974
	rs3020314					
	C/C	235 (71.0)	271 (75.1)	Reference		
	T/C	96 (29.0)	90 (24.9)	0.72 (0.49–1.04)	0.081	0.218
	rs3778082					
	G/G	129 (42.4)	164 (46.2)	Reference		
	G/A	146 (48.0)	158 (44.5)	0.70 (0.48–1.02)	0.061	0.184
	A/A	29 (9.5)	33 (9.3)	0.65 (0.34–1.22)	0.176	0.340
	rs3778099					
	T/T	37 (34.9)	137 (36.9)	Reference		
	C/T	57 (53.8)	195 (52.6)	0.84 (0.51–1.49)	0.511	0.710
	C/C	12 (11.3)	39 (10.5)	0.84 (0.37–1.86)	0.659	0.783
	rs3798573					
	A/A	126 (40.5)	157 (44.9)	Reference		
	A/G	154 (49.5)	160 (45.7)	0.70 (0.49–1.00)	0.051	0.166
	G/G	31 (10.0)	33 (9.4)	0.58 (0.31–1.08)	0.089	0.229
	rs3798577					
	C/C	72 (22.4)	52 (14.5)	Reference		
	T/C	184 (57.3)	206 (57.5)	1.48 (0.94–2.33)	0.092	0.233
	T/T	65 (20.2)	100 (27.9)	2.23 (1.32–3.78)	0.003	0.124
	rs851982					
	T/T	266 (81.6)	258 (74.1)	Reference		
	T/C	60 (18.4)	83 (23.9)	1.51 (1.00–2.27)	0.047	0.164
	C/C	0 (0.0)	7 (2.0)	NA	0.973	0.980
	rs9322331					
	C/C	315 (96.0)	336 (94.1)	Reference		
	T/C	13 (4.0)	20 (5.6)	1.31 (0.60–2.85)	0.503	0.705
	T/T	0 (0.0)	1 (0.3)	NA	0.980	0.980
	rs9340799					
	A/A	159 (48.8)	243 (69.6)	Reference		
	G/A	144 (44.2)	93 (26.6)	0.40 (0.28–0.58)	< 0.0001	<**0.0001**
	G/G	23 (7.1)	13 (3.7)	0.45 (0.20–1.00)	0.049	0.164
	rs1999805					
	G/G	167 (53.9)	140 (39.7)	Reference		
	G/A	103 (33.2)	178 (50.4)	1.91 (1.32–2.75)	0.001	**0.013**
	A/A	40 (12.9)	35 (9.9)	0.98 (0.56–1.72)	0.953	0.978
*ESR2*	rs1255998					
	C/C	90 (29.7)	104 (29.5)	Reference		
	C/G	146 (48.2)	161 (45.7)	0.99 (0.66–1.48)	0.959	0.978
	G/G	67 (22.1)	87 (24.7)	1.12 (0.70–1.80)	0.630	0.773
	rs928554					
	T/T	128 (41.7)	179 (50.0)	Reference		
	C/T	148 (48.2)	152 (42.5)	0.89 (0.63–1.27)	0.523	0.716
	C/C	31 (10.1)	27 (7.5)	0.60 (0.32–1.15)	0.124	0.280
	rs960070					
	G/G	194 (63.8)	179 (52.5)	Reference		
	G/C	99 (32.6)	141 (41.3)	1.33 (0.92–1.91)	0.125	0.280
	C/C	11 (3.6)	21 (6.2)	2.03 (0.85–4.85)	0.110	0.262
	rs1152579					
	G/G	128 (40.6)	184 (52.4)	Reference		
	G/A	154 (48.9)	141 (40.2)	0.77 (0.54–1.09)	0.141	0.299
	A/A	33 (10.5)	26 (7.4)	0.55 (0.29–1.04)	0.065	0.189
	rs944459					
	C/C	156 (49.4)	201 (56.3)	Reference		
	C/T	139 (44.0)	129 (36.1)	0.77 (0.54–1.09)	0.136	0.297
	T/T	21 (6.6)	27 (7.6)	1.06 (0.55–2.04)	0.868	0.933
*PGR*	rs471767					
	A/A	299 (90.3)	325 (90.3)	Reference		
	A/G	32 (9.7)	35 (9.7)	1.19 (0.68–2.09)	0.534	0.716
	rs484389					
	A/A	262 (79.2)	248 (70.9)	Reference		
	A/G	61 (18.4)	95 (27.1)	1.67 (1.12–2.49)	0.013	0.079
	G/G	8 (2.4)	7 (2.0)	1.14 (0.34–3.76)	0.833	0.908
	rs508653					
	C/C	267 (81.2)	264 (73.7)	Reference		
	C/A	53 (16.1)	84 (23.5)	1.84 (1.20–2.81)	0.005	0.051
	A/A	9 (2.7)	10 (2.8)	0.68 (0.25–1.88)	0.457	0.684
	rs547378					
	G/G	262 (79.2)	253 (70.7)	Reference		
	G/A	61 (18.4)	97 (27.1)	1.69 (1.14–2.53)	0.010	0.068
	A/A	8 (2.4)	8 (2.2)	1.22 (0.38–3.87)	0.735	0.836
	rs561650					
	T/T	265 (80.1)	262 (74.0)	Reference		
	T/C	64 (19.3)	85 (24.0)	1.44 (0.96–2.15)	0.077	0.216
	C/C	2 (0.6)	7 (2.0)	3.33 (0.59–18.9)	0.174	0.340
	rs566351					
	C/C	260 (79.5)	256 (72.1)	Reference		
	C/T	62 (19.0)	89 (25.1)	1.51 (1.00–2.27)	0.049	0.164
	T/T	5 (1.5)	10 (2.8)	1.46 (0.44–4.85)	0.538	0.716
	rs572483					
	T/T	263 (79.9)	259 (73.4)	Reference		
	C/T	57 (17.3)	83 (23.5)	1.70 (1.12–2.58)	0.013	0.079
	C/C	9 (2.7)	11 (3.1)	0.76 (0.28–2.05)	0.588	0.739
	rs613120					
	A/A	265 (80.1)	262 (73.8)	Reference		
	A/G	57 (17.2)	82 (23.1)	1.66 (1.09–2.52)	0.017	0.092
	G/G	9 (2.7)	11 (3.1)	0.75 (0.28–2.01)	0.561	0.734
	rs500760					
	T/T	262 (79.2)	247 (70.6)	Reference		
	C/T	61 (18.4)	95 (27.1)	1.63 (1.09–2.44)	0.017	0.092
	C/C	8 (2.4)	8 (2.3)	1.25 (0.39–3.99)	0.702	0.810
	rs608995					
	A/A	258 (78.9)	244 (69.7)	Reference		
	T/A	61 (18.7)	98 (28.0)	1.71 (1.15–2.56)	0.008	0.063
	T/T	8 (2.4)	8 (2.3)	1.24 (0.39–3.93)	0.711	0.815
*PPARG*	rs1801282					
	C/C	271 (83.4)	321 (89.2)	Reference		
	C/G	54 (16.6)	39 (10.8)	0.56 (0.35–0.91)	0.020	0.095
	rs709149					
	G/G	95 (30.0)	132 (37.9)	Reference		
	G/A	177 (55.8)	176 (50.6)	0.73 (0.50–1.07)	0.108	0.261
	A/A	45 (14.2)	40 (11.5)	0.86 (0.48–1.54)	0.621	0.768
	rs709151					
	C/C	85 (26.2)	133 (37.6)	Reference		
	T/C	189 (58.3)	182 (51.4)	0.61 (0.42–0.89)	0.009	0.067
	T/T	50 (15.4)	39 (11.0)	0.60 (0.34–1.04)	0.069	0.197
	rs709154					
	A/A	90 (27.5)	156 (43.9)	Reference		
	T/A	187 (57.2)	160 (45.1)	0.47 (0.33–0.69)	< 0.0001	<**0.0001**
	T/T	50 (15.3)	39 (11.0)	0.54 (0.31–0.94)	0.030	0.124
	rs1151996					
	A/A	88 (27.2)	125 (35.9)	Reference		
	C/A	154 (47.7)	182 (52.3)	0.81 (0.55–1.19)	0.285	0.457
	C/C	81 (25.1)	41 (11.8)	0.40 (0.24–0.68)	0.010	0.068
*LAMC1*	rs1413390					
	A/A	97 (30.5)	88 (25.4)	Reference		
	G/A	174 (54.7)	195 (56.4)	1.14 (0.77–1.70)	0.501	0.705
	G/G	47 (14.8)	63 (18.2)	1.43 (0.85–2.40)	0.184	0.344
	rs1537520					
	T/T	97 (29.5)	88 (24.8)	Reference		
	C/T	185 (56.2)	206 (58.0)	1.15 (0.78–1.71)	0.473	0.684
	C/C	47 (14.3)	61 (17.2)	1.44 (0.85–2.44)	0.174	0.34
	rs20558					
	C/C	96 (29.2)	88 (24.6)	Reference		
	T/C	187 (56.8)	208 (58.3)	1.12 (0.75–1.65)	0.585	0.739
	T/T	46 (14.0)	61 (17.1)	1.42 (0.84–2.41)	0.192	0.346
	rs2296293					
	A/A	97 (30.1)	87 (25.0)	Reference		
	A/G	183 (56.8)	201 (57.8)	1.13 (0.76–1.69)	0.536	0.716
	G/G	42 (13.0)	60 (17.2)	1.67 (0.97–2.86)	0.065	0.189
	rs2296300					
	A/A	97 (29.3)	89 (25.0)	Reference		
	A/G	187 (56.5)	206 (57.9)	1.12 (0.76–1.66)	0.570	0.735
	G/G	47 (14.2)	61 (17.1)	1.41 (0.83–2.39)	0.199	0.351
	rs6424888					
	G/G	97 (29.3)	88 (24.6)	Reference		
	G/A	187 (56.5)	207 (58.0)	1.12 (0.76–1.66)	0.559	0.734
	A/A	47 (14.2)	62 (17.4)	1.42 (0.84–2.40)	0.190	0.346
	rs6672093					
	C/C	90 (27.9)	83 (23.2)	Reference		
	T/C	181 (56.0)	205 (57.4)	1.09 (0.73–1.64)	0.661	0.783
	T/T	52 (16.1)	69 (19.3)	1.35 (0.80–2.26)	0.261	0.431
*HMGA2*	rs2272046					
	A/A	227 (68.6)	320 (88.9)	Reference		
	C/A	104 (31.4)	40 (11.1)	0.28 (0.18–0.43)	< 0.0001	<**0.0001**
*MTHFR*	rs1801133					
	C/C	96 (31.3)	52 (15.5)	Reference		
	T/C	139 (45.3)	162 (48.2)	1.88 (1.19–2.96)	0.007	0.061
	T/T	72 (23.5)	122 (36.3)	2.64 (1.60–4.34)	< 0.0001	<**0.0001**
*SUMO1P1*	rs6022786					
	G/G	112 (35.0)	167 (47.7)	Reference		
	G/A	169 (52.8)	143 (40.9)	0.58 (0.41–0.84)	0.046	0.160
	A/A	39 (12.2)	40 (11.4)	0.69 (0.39–1.21)	0.194	0.346

*Boldface indicates P < 0.05; CI, confidence interval; NA, not available; OR, odds ratio; PCOS, polycystic ovarian syndrome; SNP, single-nucleotide polymorphism*.

a *P value was from unconditional logistic regression with adjustment for BMI and age*.

*P_ajusted_ were performed for each gene using Bonferroni correction*.

### Haplotype block structure and haplotype analysis

**Figure 1:**
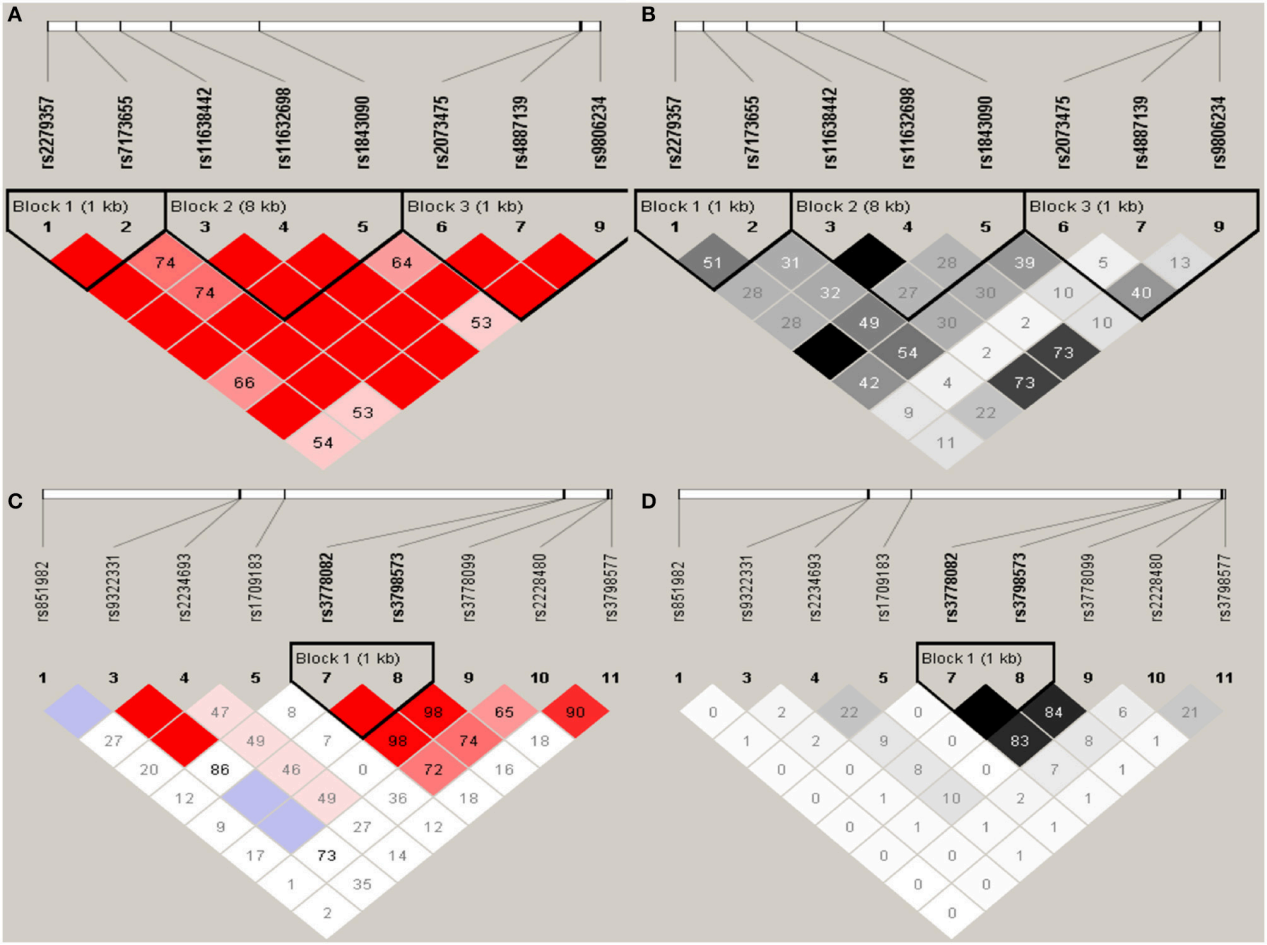
Graphical representations of the SNP locations and LD structure of *CYP11A* **(A,B)** and *ESR1* **(C,D)** using tested genotyped SNPs in 331 controls.

**Figure 2:**
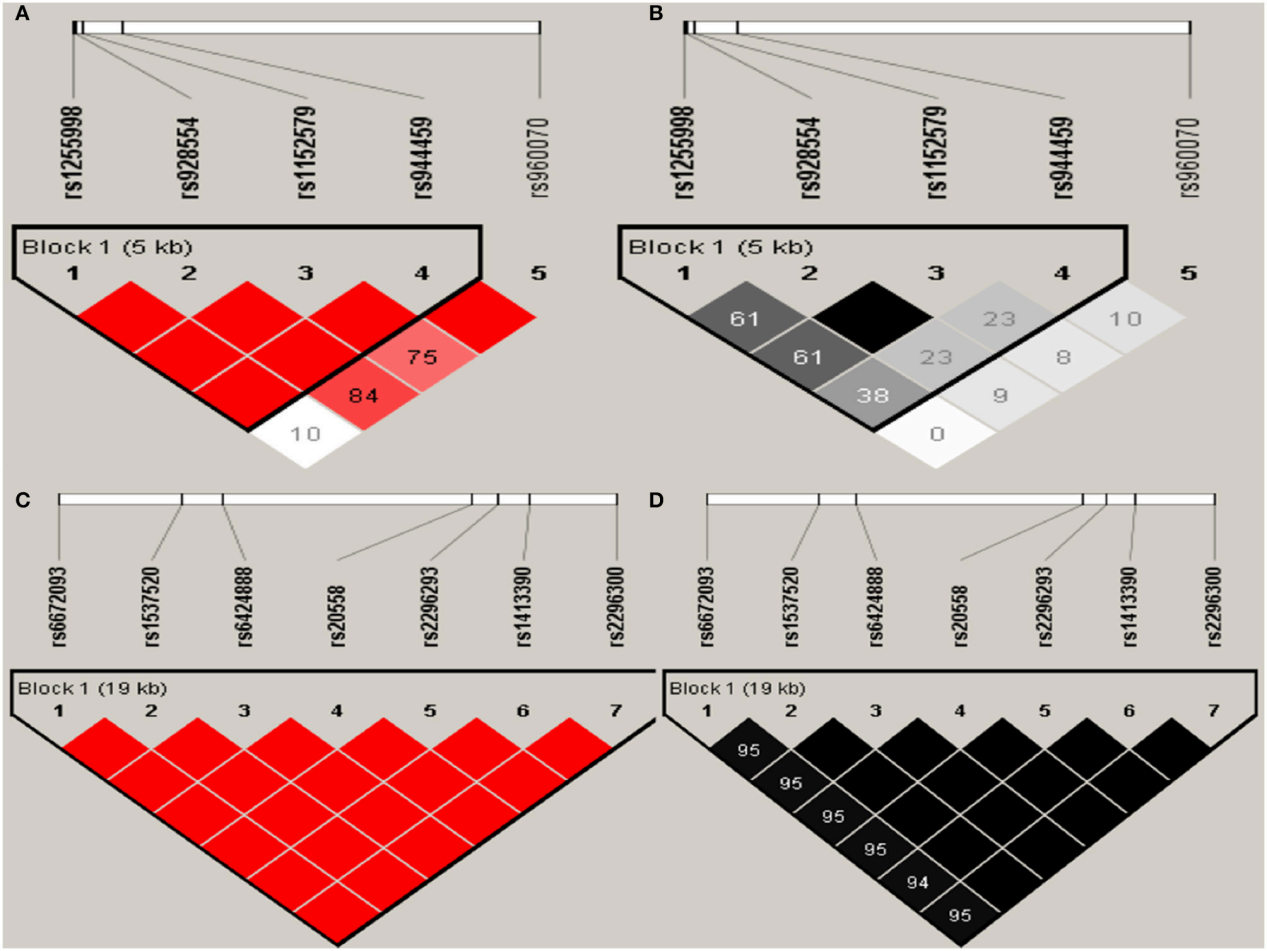
Graphical representations of the SNP locations and LD structure of *ESR2* **(A,B)** and *LAMC1* **(C,D)** using tested genotyped SNPs in 331 controls.

**Figure 3:**
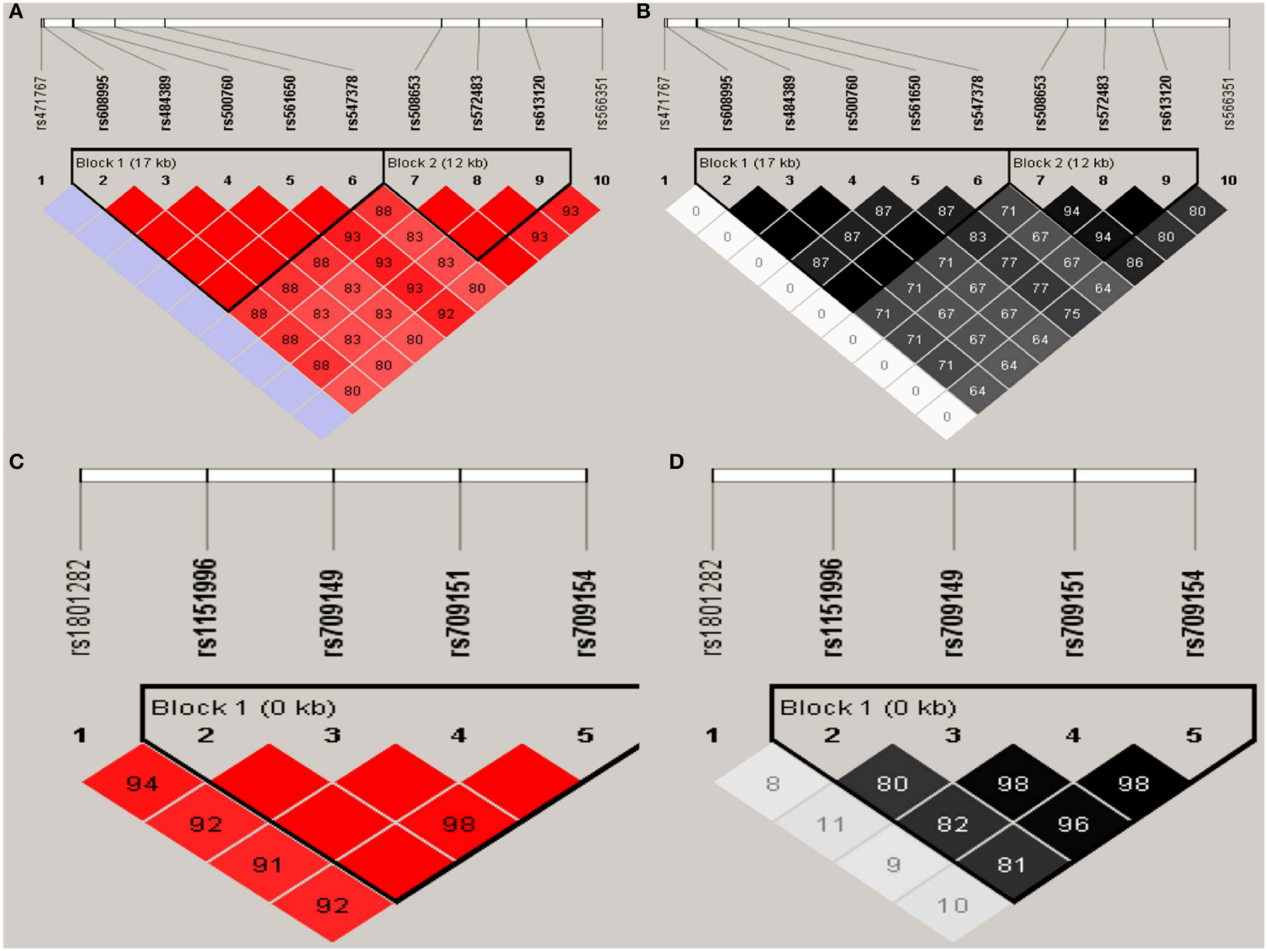
Graphical representations of the SNP locations and LD structure of *PGR* **(A,B)** and *PPARG* **(C,D)** using tested genotyped SNPs in 331 controls.

Haploview analysis indicated strong LD among SNPs in the genes *CYP11A1, ESR1, ESR2, LAMC1, PGR*, and *PPARG* (Figures 1–3). In *CYP11A1*, three haplotype blocks were defined using 8 genotyped SNPs. In *PGR*, two haplotype blocks were formed: the first spanned 17 kb and contained five tested SNPs, and the second contained three tested SNPs. In addition, *ESR1, ESR2, LAMC1*, and *PPARG* were constructed respectively with one block. Table 5 summarizes the associations between genetic haplotypes and PCOS risk. After adjusting for age and BMI, the risk of PCOS was significantly increased among individuals carrying the haplotype “CGA” in Block3 of *CYP11A1* (OR = 2.30, 95% CI = 1.45–3.66, *P*_ajusted_ = 0.013), compared with those carrying the most common haplotype “TAG.” Similarly, the risk was also increased among individuals carrying the haplotype “GTGC” in *ESR2* (OR = 2.01, 95% CI = 1.33–3.02, *P*_ajusted_ = 0.020), compared with those carrying the haplotype “GCAC.” Furthermore, one highly protective haplotype “CGCA” in *PPARG* with 87% reduction in risk of developing PCOS comparing with those carrying the haplotype “AGCA” (OR = 0.13 and 95% CI = 0.04–0.40, *P*_ajusted_ = 0.010).

**Table 5:** Association between *CYP11A1, ESR1, ESR2, LAMC, PGR*, and *PPARG* genetic haplotypes and PCOS risk.

**Gene**	**Haplotype^a^**	**Frequencies**		**Logistic regression**		
		**Controls**	**Cases**	**OR (95% CI)^b^**	***P***	***P_*ajusted*_***
*CYP1A11*						
Block1	CT	0.412	0.412	1.00 (reference)	–	–
	TC	0.421	0.403	0.96 (0.73–1.27)	0.779	0.874
	CC	0.167	0.184	1.15 (0.81–1.64)	0.430	0.643
Block2						
	CAA	0.415	0.395	1.00 (reference)	–	–
	CAG	0.298	0.354	1.22 (0.92–1.62)	0.159	0.324
	GGG	0.287	0.251	0.95 (0.70–1.28)	0.702	0.810
Block3						
	TAG	0.437	0.394	1.00 (reference)	–	–
	CAA	0.281	0.242	1.06 (0.77–1.45)	0.702	0.810
	CAG	0.219	0.25	1.29 (0.94–1.79)	0.191	0.346
	CGA	0.063	0.113	2.30 (1.45–3.66)	5*10^4^	**0.013**
*ESR1*	GA	0.654	0.684	1.00 (reference)	–	–
	AG	0.346	0.317	0.72 (0.55–0.93)	0.013	0.079
*ESR2*	GCAC	0.356	0.278	1.00 (reference)	–	–
	CTGT	0.291	0.256	1.15 (0.84–1.57)	0.380	0.593
	CTGC	0.234	0.26	1.34 (0.97–1.86)	0.074	0.214
	GTGC	0.119	0.199	2.01 (1.33–3.02)	9*10^4^	**0.020**
*LAMC1*	CTGCAAA	0.563	0.519	1.00 (reference)	–	–
	TCATGGG	0.425	0.462	1.20 (0.93–1.56)	0.170	0.340
	TTGCAAA	0.013	0.015	1.07 (0.38–3.03)	0.900	0.955
*PGR*						
Block1	AATTG	0.884	0.838	1.00 (reference)	–	–
	TGCCA	0.103	0.142	1.52 (1.06–2.20)	0.025	0.101
	TGCTA	0.014	0.015	1.12 (0.41–3.04)	0.830	0.908
Block2	CTA	0.887	0.85	1.00 (reference)	–	–
	ACG	0.107	0.144	1.35 (0.95–1.90)	0.090	0.233
*PPARG*						
	AGCA	0.515	0.616	1.00 (reference)	–	–
	CATT	0.429	0.325	0.77 (0.59–1.00)	0.053	0.166
	CGCA	0.048	0.006	0.13 (0.04–0.40)	4*10^4^	**0.010**
	CATA	0.003	0.034	10.89 (2.32–51.19)	0.003	0.124

*Boldface indicates P < 0.05, CI, confidence interval; OR, odds ratio*.

a *Polymorphic bases are listed in 5′-3′ order as in Figure 1*.

b *Adjusted for age and BMI. P_ajusted_ were performed for each gene using Bonferroni correction*.

In *CYP11A1*, three haplotype blocks were defined using 8 genotyped SNPs. In *ESR1*, one haplotype block was defined in each gene using the Haploview program with default settings. The confidence interval minima were upper 0.98 and low 0.7 in the case of strong LD; the upper confidence interval maximum was 0.9 for strong recombination; and the fraction of strong LD in informative comparisons had to be at least 0.95. SNPs are indicated by rs number (top: from left to right). (A) The numbers in squares are D′ values (|D′| × 100). The measure of LD (D′) among all possible pairs of SNPs is shown graphically according to the shade of red, where white represents very low D′ and dark red represents very high D′. (B) The measure of LD (*r*^2^) among all possible pairs of SNPs is shown graphically according to the shade of gray, where white represents very low *r*^2^ and black represents very high *r*^2^.

In *ESR2* and *LAMC1*, one haplotype block was defined in each gene using the Haploview program with default settings.

In *PGR*, two haplotype blocks were defined using 10 genotyped SNPs. In *PPARG*, one haplotype block was defined in each gene using the Haploview program with default settings.

## Discussion

In this PCOS case-control study in a Chinese Han population, we found the following genotypes were associated with a lower risk of developing PCOS: GA genotype of rs9340799 in *ESR1*, TA genotype of rs709154 in *PPARG*, and CA genotype of rs2272046 in *HMGA*. Conversely, the GA genotype of rs1999805 in *ESR1*, and the TT genotype of rs1801133 in *MTHFR* were significantly associated with increased risk of PCOS. Associations of *ESR1* rs9340799 (25), *HMGA2* rs2272046 (21), and *MTHFR* rs1801133 (32) with PCOS risk have been reported in other studies. In contrast, our results appear to provide the first evidence linking rs1999805 in *ESR1* and rs709154 in *PPARG* with PCOS risk in a Chinese population. These findings provide evidence that polymorphism in genes involved in hormonal action, lipid metabolism and insulin action may modify PCOS risk.

Polymorphism in the *ESR1* gene may influence PCOS risk because the protein is necessary for the proper functioning of the hypothalamic-pituitary-ovarian axis. The protein is up-regulated in theca cells of polycystic ovaries, such that the ratio of *ESR1* to *ESR2* expression is elevated in PCOS, which may contribute to abnormal follicular development (33). Whatever the underlying mechanism, the association between *ESR1* polymorphism and risk of PCOS appears to depend in complex ways on ethnicity. A Pakistani study linked the CC genotype of *ESR1* rs2234693, the GG genotype of rs9340799, and the CT genotype of rs8179176 with elevated disease risk (25). However, studies in Greeks (34) and Caucasians (35) revealed no differences in frequencies of these genotypes between PCOS patients and controls. The present study found that the GA genotype of rs9340799 protected Chinese women from PCOS. Further study should clarify to what extent these divergent findings reflect ethnicity.

Our results raise the possibility of a correlation between polymorphism at rs1999805 in influencing PCOS risk. We found that the GA genotype of rs1999805 increased that risk in a Chinese population. Since rs1999805 lies in an *ESR1* intron, the question arises whether the polymorphism contributes to disease or is simply a marker in LD with other untyped functional variants. Our LD analysis does not predict other disease-relevant sites in this gene. Therefore, the pathway(s) connecting rs1999805 polymorphism with PCOS warrant further exploration.

Our finding of a link between rs709154 and rs1151996 in *PPARG*, which lies in an intron of the gene, and risk of PCOS may be explained by the fact that the encoded protein functions as a nuclear hormone receptor to play a crucial role in lipogenesis, cell differentiation, inflammatory cytokine production, glucose homeostasis and insulin sensitization (36). We found evidence that *PPARG* rs709154 and rs1151996 acts as a protective factor against PCOS. Similarly, a meta-analysis has concluded that *PPARG* rs1801282 C>G polymorphism is associated with decreased PCOS risk. Conversely, the *PPARG* rs1801282 (Pro12Ala) polymorphism increased PCOS risk in a study comparing 100 African PCOS patients and 120 healthy controls (37). We found *PPARG* rs709154 to be in strong LD with rs709149 and rs709151, but we are unaware of studies investigating possible correlation among these three polymorphisms in PCOS risk. Future work should examine this question.

Our results with rs2272046 in *HMGA2* are consistent with those of a previous study of 744 Han Chinese PCOS patients and 895 healthy controls linking the SNP with decreased PCOS risk (21). The polymorphism rs2272046 is located in an *HMGA2* intron, raising the possibility that it affects risk of PCOS by altering degradation or translation of the *HMGA2* mRNA (38). The SNP rs2272046 is in complete LD with rs74980477 (D′ = 1, *r*^2^ = 1), and the latter is predicted to alter the function of the myc gene. Normal expression of myc oncoprotein plays a critical role in initial oocyte growth and autonomous growth of granulosa cells, so dysregulation may affect follicular development (39). However, This study didn't revealed that *SUMO1P1* was associated with PCOS risk, and the result was inconsistent with the GAWS studies (21). The population geographic differences, our limited power and clinical features differences in these subjects may partly explain the inconsistent results.

The results with rs1801133 in *MTHFR* are consistent with an Iranian study linking the CC genotype at this locus with decreased PCOS risk (32). The SNP rs1801133 occurs in an *MTHFR* exon that changes an Ala to Val, based on South Han Chinese data from the 1000 Genomes Project (40). This polymorphism is associated with acetylation of lysine 27 of the H3 histone protein, and this H3K27Ac mark is believed to enhance transcription, possibly by blocking the spread of the repressive histone mark H3K27Me3, which is usually found near regulatory elements and is considered to enhance transcription (41). In this way, polymorphism at rs1801133 may affect *MTHFR* expression and thereby affect risk of PCOS.

This study has some limitations. Our relatively small sample size limited the statistical power of our findings. We recruited only Han Chinese participants, preventing the analysis of how our SNP results depend on ethnicity. The fact that five of our 63 SNPs deviated from HWE and 5 had a minor allele frequency of 0 greatly reduced the rate of locus detection. Since our study lacked a validation group, further work is needed to confirm our observed associations between SNPs and PCOS risk.

In summary, we found, the first evidence that polymorphism at *ESR1* rs1999805 and *PPARG* rs709154 were significantly associated with PCOS risk in Han Chinese. GA genotype at rs9340799 in *ESR1*, TA genotype at rs709154 in *PPARG*, CA genotype at rs2272046 in *HMGA2*, and GA genotype at rs6022786 in *SUMO1P1* may be associated with a lower risk of developing PCOS. Conversely, GA genotype at rs1999805 in *ESR1*, TT genotype at rs1801133 in *MTHFR* correlated with elevated PCOS risk. Our findings may help clarify the genetics of PCOS and generate leads for further functional research on the disease. Further investigations with larger, multi-ethnic samples are required to confirm our results.

## Author contributions

XJ contributed significantly to analysis and wrote the manuscript. JZ and WW conceived and designed the experiments. JS and DC helped perform the analysis with constructive discussions. WC, YS, and WZ helped collect data and test specimens. XY designed the experiments and approved the final version.

### Conflict of interest statement

The authors declare that the research was conducted in the absence of any commercial or financial relationships that could be construed as a potential conflict of interest.
