# Supplementary material for: Variant Alleles of the ESR1, PPARG, HMGA2, and MTHFR Genes Are Associated With Polycystic Ovary Syndrome Risk in a Chinese Population: A Case-Control Study
Source: Front Endocrinol (Lausanne). 2018 Aug 30;9:504. doi: 10.3389/fendo.2018.00504 (PMC6125305; doi:10.3389/fendo.2018.00504)
Supplement: Supplementary file 1 [file Table_1.DOCX]

**Supplementary Table1．**Stratified analysis of associations between the related polymorphisms in PCOS patients and controls by BMI

|  | BMI | | | | | |
| --- | --- | --- | --- | --- | --- | --- |
| Gene | SNP | BMI < 24 | | BMI ≥ 24 | | *P* interaction |
| *ESR1* | rs9340799 | OR (95%CI) | *P^a^* | OR (95%CI) | *P^a^* |  |
|  | GA vs. AA | 0.32 (0.21, 0.50) | **<0.0001** | 0.81 (0.40, 1.63) | 0.547 | 0.053 |
|  | GG vs. AA | 0.49 (0.21, 1.18) | 0.112 | 0.29 (0.07, 1.16) | 0.08 |  |
|  | rs1999805 |  |  |  |  |  |
|  | GA vs. GG | 2.34 (1.53, 3.59) | **<0.0001** | 1.25 (0.64, 2.45) | 0.507 | 0.151 |
|  | AA vs. GG | 0.81 (0.40, 1.64) | 0.559 | 1.24 (0.42, 3.68) | 0.694 |  |
| *PPARG* | rs709154 |  |  |  |  |  |
|  | TA vs. AA | 0.53 (0.34, 0.81) | **0.003** | 0.28 (0.12, 0.62) | **0.002** | 0.32 |
|  | TT vs. AA | 0.49 (0.27, 0.91) | **0.025** | 0.46 (0.13, 1.67) | 0.238 |  |
| *HMGA2* | rs2272046 |  |  |  |  |  |
|  | CA vs. AA | 0.27 (0.16, 0.46) | **<0.0001** | 0.24 (0.11, 0.51) | **0.000** | 0.826 |
| *MTHFR* | rs1801133 |  |  |  |  |  |
|  | TC vs. CC | 2.02 (1.18, 3.46) | **0.010** | 2.10 (0.95, 4.67) | 0.068 | 0.851 |
|  | TT vs. CC | 3.00 (1.69, 5.35) | **0.000** | 4.03 (1.51, 10.76) | **0.006** |  |

Bold characters indicate that corresponding *P* value is less than 0.05.

CI, confidence interval; OR, odds ratio; SNP, single-nucleotide polymorphism;

BMI, body mass index.

^a^*P* value from unconditional logistic regression analysis with adjustment for age.
